# Identification and mapping of quantitative trait loci for resistance to *Liriomyza trifolii* in romaine lettuce cultivar ‘Valmaine’

**DOI:** 10.1038/s41598-020-80050-5

**Published:** 2021-01-13

**Authors:** Ramkrishna Kandel, Huangjun Lu, Germán V. Sandoya

**Affiliations:** 1 Horticultural Sciences Department, University of Florida, Gainesville, FL 32611 USA; 2 Everglades Research and Education Center, Institute of Food and Agricultural Sciences/University of Florida, Belle Glade, FL 33430 USA

**Keywords:** Agricultural genetics, Genetic linkage study, Genetic markers, Plant breeding, Plant genetics, Plant sciences

## Abstract

*Liriomyza trifolii* (Diptera: Agromyzidae) is a leafminer that causes ruinous damage to many leafy vegetables including lettuce (*Lactuca sativa* L.) by stippling and tunneling the leaves. In this study, a population of 125 F_3_ families was developed from the intraspecific cross of ‘Valmaine’ (resistant) and ‘Okeechobee’ (susceptible) romaine cultivars for inheritance analysis and molecular mapping of the resistance loci controlling stippling damage. The experiments were conducted in an insectarium (controlled environment). Stippling damage proved to be heritable because the broad-sense heritability (*H*^2^) was 0.58. A segregation analysis suggested that a single dominant allele, *Sd1 locus,* controls resistance against *L. trifolii*. Furthermore, a quantitative trait loci (QTL) analysis identified one novel QTL, named Stippling on LG5 (*qSTP5*), flanked by two SNPs that were mapped to a 5.2 cM (8.5 Mb region) interval, explaining over 13% of the total phenotypic variance. Desirable allele for resistance to *L. trifolii* was derived from resistant cultivar Valmaine. Identification of SNPs closely linked to the QTL responsible for *L. trifolii* resistance should facilitate plant breeders to develop resistant romaine lettuce cultivars.

## Introduction

Lettuce is one of the most important vegetable crops grown in the United States in terms of production and consumption^1^. In the U.S., lettuce is frequently attacked by several insect pests, one of which is *L. trifolii*^2–4^. Besides lettuce, this polyphagous species^5^ attacks as many as 25 plant families with preference on the Asteraceae^6^ where lettuce belongs. This pest causes major economic losses in both ornamental and vegetables crops such as Chrysanthemums^7^ and celery^8^, and onion^9^. The unsightly appearances caused by larval mines reduce the value of ornamental and vegetables^10,11^. For instance, the estimated economic losses of celery caused by *L. trifolii* is reported to be US $9 million in 1980^8^; the losses of celery primarily resulted from cosmetic and yield reduction^12^. Yield losses in lettuce due to *L. trifolii* damage is twofold. First, reduced photosynthesis caused by stippling (scars from feeding and oviposition punctures) and tunneling (mines developed from larval feeding) of leaves. Second, unmarketability due to unsightly appearance of stipples. A loss to the lettuce industry due to leafminer damage was estimated to be $1 million annually in south Florida alone^3^.

*Liriomyza trifolii* outbreaks have occurred in Florida since the late 1940s^13^ and in Arizona and California since the late 1980s^14^. Chemical insecticides are used to control *L. trifolii*^15^ even though the evidence of insecticide resistance in *Liriomyza* spp. in Florida was documented during the early 1950s^16^. The failure of insecticides to control *L. trifolii* led to the near collapse of the Florida celery industry^17^. An integrated pest management program was then implemented as a judicious approach to manage the insecticide resistance problem^18,19^. Use of insecticides to control these pests has been ineffective economically as well as ecologically^20^. Although natural enemies of *L. trifolii* such as *Diglyphus isaea* (Walker) (Hymenoptera: Eulophidae) and *Dacnusa sibirica* Telegna (Hymenoptera: Braconidae) are effective in cucumber and tomato^21^, their effectiveness has not been documented in lettuce.

Information on genetic variation^2^, stability, and mechanism of resistance (both antixenotic—non-host preference of the insect because of physical structure of a plant- as well as antibiotic—when a plant possesses deleterious effects on the insect) to *Liriomyza* spp. has been reported in cultivars and wild germplasm^22,23^. Resistance sources have been incorporated into elite lettuce cultivars for California production^23^, but not for Florida production because of the existence of efficacious insecticides to control this pest in Florida, and also diseases caused by many microorganisms are more detrimental to lettuce production. However, insecticide resistance may make chemical control ineffective or current insecticide labels may be phased out. Therefore, host plant resistance must be considered as an approach within an Integrated Pest Management to control *L. trifolii*. Host plant resistance combined with other tactics for controlling polyphagous pest species is known to be the most effective strategy^24^.

To design efficient breeding strategies for *L. trifolii* resistance, it is important to understand the inheritance of the resistance trait. However, there is a complete lack of information on the underlying genetics of *L. trifolii* resistance and molecular mapping of the resistance loci in lettuce. Thus, the objectives of this study were to (i) determine the mode of *L. trifolii* resistance in romaine lettuce and (ii) map the underlying resistance loci.

## Materials and methods

### Mapping population

A cross between the resistant cultivar Valmaine (PI 543959) and the susceptible Okeechobee (PI 658142) was made to study the inheritance of the resistance to insect pests in lettuce*.* The resulting F_1_ was planted in greenhouse conditions to produce F_2_ seeds by self-pollination. Approximately 125 F_2_ individual plants were randomly chosen to develop the F_3_ families used in this study. The F_3_ families used to perform progeny test in this research were derived from the same F_2_ mapping population used by^25^ to study the genetics of lettuce against *Diabrotica balteata*.

### F_3_ family phenotyping

#### Establishment and maintenance of *Liriomyza trifolii* colony

A *Liriomyza trifolii* colony was established from adults collected from a lettuce field in Belle Glade, FL and maintained on cowpea seedlings, *Vigna unguiculata* L in the insectarium. The room temperature and photoperiod were maintained at 27 °C with 24 h of light, respectively. One-week-old cowpea seedlings were transferred to a wooden cage 45 × 45 × 60 cm every day and newly emerged adult *L. trifolii* were released. Cowpea seedlings were then transferred to a tray the following day and watered adequately to allow plant growth and larvae development. After two days, seedlings were transferred to a vertical wooden structure that supported the trays horizontally to facilitate the collection of pupae as they emerged from leaves. After four days of collection, pupae were put into a vial and stored in the same room until adult emergence.

#### Greenhouse test

Because lettuce is a winter crop in South Florida, we were able to conduct the experiments from September through April in 2013 and the same in 2014. Experiments were conducted in greenhouse only because there is not a method to infest *L. trifolii* in field settings. Relying on natural infestations was not possible as insecticides are constantly applied in fields nearby. Plants were grown in greenhouse for about seven weeks and then moved to the insectary room maintained at 27 °C and a photoperiod of 14:10 (L:D) h. Plants were watered and fertilized adequately while kept for two days in the insectary room for *L. trifolii* testing. Plants were transferred to a greenhouse after the *L. trifolii* test and allowed to grow until seed maturity.

Fully expanded leaves from the middle to upper portion of the plants (i.e. leaves in positions 5 to 9 numbered starting from the first true leaf at the bottom of the plants) were selected for *L. trifolii* greenhouse experiments^2^ because *L. trifolii* prefers these leaves over both the older and younger leaves. A no-choice test was performed to assess the reaction of plants to insect feeding. A clip cage (radius = 1.9 cm, height = 2.8 cm) was attached to a leaf of an individual plant supported by a bamboo stick. Due to the difficulties in determining the sex of *L. trifolii* accurately and quickly, we selected mating pairs to ensure the number of female flies at least 50% in each clip cage. Two mating pairs of *L. trifolii* less than 24 h old were aspirated into a glass tube and released into each clip cage. The flies were allowed to feed on a leaf for 48 h.

#### Experimental design

The trial was conducted using two replications arranged as a randomized complete block design (RCBD). On each replication, six plants of each of the 20 F_3_ families were grown in 15-cm pots. In addition, six plants per replication for each parent ‘Okeechobee’ and ‘Valmaine’ were planted as susceptible and resistant controls. Due to highly tedious work involved in *L. trifolii* colony maintenance, mating pair collection, and stippling damage evaluation, the experiments were conducted using two replications.

#### Trait assessment

**Figure 1:**
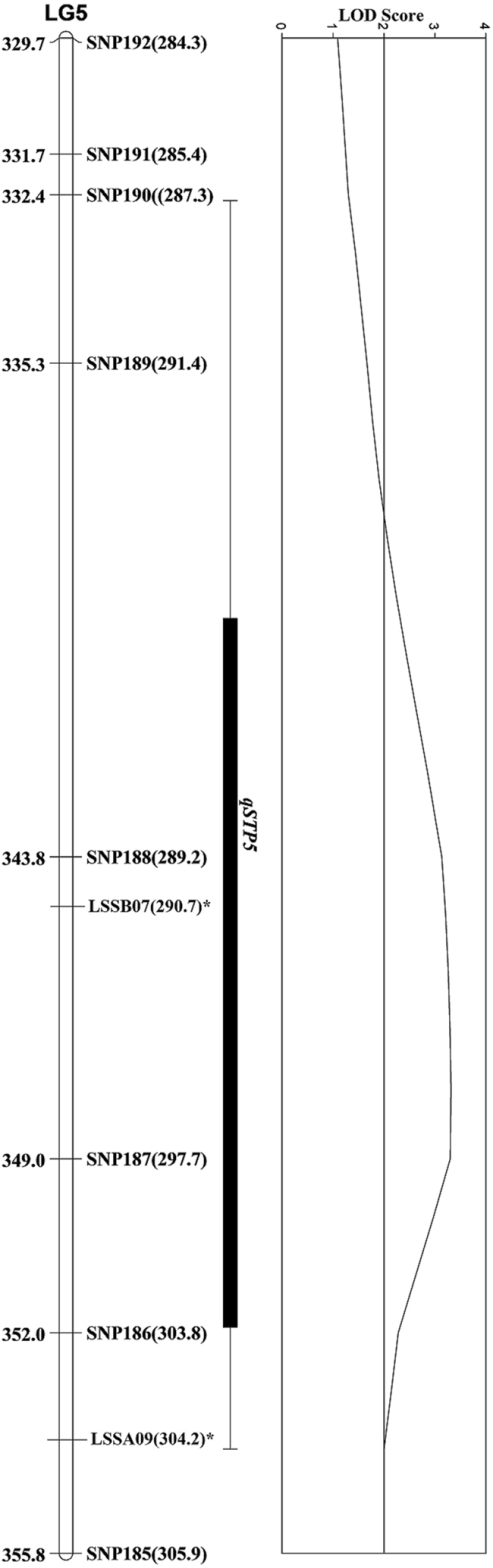
A section of LG5 delineating genomic location of QTL *qSTP5.* Map distances are shown in cM on the left of linkage group, while genetic markers are on the right of linkage group. QTL position is shown on the bars right to linkage group. In brackets are physical positions (Mb) of each marker. In asterisks are the SSRs that were used as anchors from Rauscher and Simko^29^. Physical positions of both SNPs and SSRs were obtained by blasting sequences against the lettuce whole genome^32^.

Forty-eight hours later, stippling damage was recorded on a rating scale of 0 to 4, where 0 = 0–20 stipples, 1 = 21–75 stipples, 2 = 76–150 stipples, 3 = 151–250 stipples, and 4 > 250. Rating scale (0–4) was developed based on previous observations of the stippling damage in the resistant and susceptible parents (Supplementary file 1; Fig. 1).

Because this was a no-choice test, resistant parent Valmaine had few stippling damages, sometimes equivalent to rating of 2. Therefore, plants with ratings of 2 or less were considered resistant, whereas plants with ratings of 3 or 4 were considered susceptible.

### Statistical analyses

#### Testing one-locus and two-locus models for stippling damage resistance

A total of 12 plants from each of the 125 F_3_ families were scored as resistant or susceptible plants. An F_3_ family was classified as homozygous resistant (A) if all plants were resistant (rating 0 to 2), homozygous susceptible (B) if all plants were susceptible (rating 3 to 4), and heterozygous (H) if both resistant and susceptible plants were identified (Supplementary file 2). Additionally, number of resistant and susceptible F_3_ plants from heterozygous (H) F_3_ families were counted to confirm if *L. trifolii* resistance was dominant to susceptibility.

We subjected the data to a chi-square (${X}^{2}$) goodness of fit test for one-locus (3:1 or 1:2:1) and the two-locus models with a complementary gene action (9:7) and a recessive suppressor (13:3). One-locus model was tested for both genotypic and phenotypic ratios, whereas two-locus model was tested only for phenotypic ratios (Table 1).

**Table 1:** Chi-square (${X}^{2}$) goodness-of-fit test for *L. trifolii* resistance using one-locus and two-locus models in romaine lettuce.

Generation	Number of individuals	Phenotype/genotype	Observed	One-locus model			Two-locus model		
				Expected	${X}^{2}$	*p* value	Expected	${X}^{2}$	*p* value
F_2_	125	R	86	93.75	2.56	0.10	70.31	8.003	0.0047†
		S	39	31.25			54.69		
							101.56	12.713	0.0004‡
							23.44		
F_3_	125	A	21	31.25	5.38	0.067		N/A	
		H	65	62.50					
		B	39	31.25					
	65	R	541	564	3.75	0.053	423	75.239	0.0001†
		S	211	188			329		
							611	42.771	0.0001‡
							141		

R, resistant; S, susceptible; A, homozygous resistant; H, heterozygous; B, homozygous susceptible.

One-locus dominant model was tested for both genotypic (1:2:1) and phenotypic (3:1) ratios.

^†^Complementary gene action (9:7).

^‡^Recessive suppressor (13:3).

#### *F*_*3*_* family genetic variation*

The 125 F_3_ families were analyzed as RCBD using PROC MIXED in SAS software^26^. In the model, plant was used as the random factor to detect differences among families; also plant and families were used as the random factor to obtain the empirical best linear unbiased predictor (EBLUPs) for posterior QTL analysis. Differences among families were calculated using the Least Square Means with Adjusted Tukey t-test at 95% level.

Additionally, a joint analysis of the 125 F_3_ families with the parents; cultivars Valmaine and Okeechobee was conducted as RCBD to detect significant differences between the F_3_ families and both parents. We used the Dunnett’s test between the F_3_ families and each of the parents to test the assumption that an F_3_ family could be significantly less damaged compared to cultivar Valmaine (the resistant parent). An F_3_ family significantly better than Valmaine is thus considered a “transgressive segregant”.

#### Estimation of genetic parameters

The PROC MIXED with ‘ASYCOV’ statement in SAS software^26^ was used to compute estimates of variance components, their associated standard errors (SEs), broad-sense heritability (*H*^2^), and their associated SEs. Individual families and plants were used as random factors. The *H*^2^ estimate of each trait was computed according to^27^ using the formula: *H*^2^ = $\frac{{\sigma }^{2}G}{[{\sigma }^{2}G+{(\sigma }^{2}/r)] }$, where σ^2^ G and σ^2^ are variance components of genotype and experimental error, respectively, and r is the number of replicates.

### Genotypic analyses

#### Genomic DNA extraction

Genomic DNA was isolated^28^ from 125 F_2_ individual plants that produced the 125 F_3_ families. The quality of genomic DNA was checked by electrophoresis in 1% agarose gel. DNA was quantified in a NanoDrop ND-1000 Spectrophotometer (Thermo Fisher Scientific, Wilmington, DE).

#### Genotyping-by-sequencing (GBS)

Genomic DNA was sent to Data2Bio, LLC (Ames, Iowa) for GBS and SNP discovery. Data2Bio used the tunable genotyping-by-sequencing (tGBS) technology to sequence the parents and F_2_ plants, identify SNPs, and genotype the entire population with the SNPs. Briefly, genomic DNA samples were digested with two restriction endonucleases (RE; N*sp*I and B*fu*CI), followed by a single-stranded barcode oligonucleotide (oligo A) ligation in one site, while the other site was ligated with another single-stranded oligonucleotide (Oligo B) complementary to amplification primer. The RE N*sp*I and B*fu*CI recognize a degenerate 5 bp sequence; *RCATG* where R is A or G, and 4 bp sequence; GATC, respectively. The low quality and redundant SNPs were deleted according to error tolerance rate ≤ 3%.

#### Simple sequence repeat (SSR) analysis

At the outset of this study, whole genome sequence information was lacking publicly. Thus, SSR markers were used in the study to anchor linkage groups to prior studies. A total of 42 genomic SSR primer pairs from Rauscher and Simko^29^ were tested for parental screening following the protocol in microsatellite applications manual^30^. A regular reverse primer, a M13 forward-tailed primer (5′—CACGACGTTGTAAAACGAC—3′), and a M13 forward-labeled primer were used for amplification and subsequent visualization of polymerase chain reaction (PCR) products. A total of seven SSRs primer pairs (17%) out of 42 primer pairs tested were added to the genetic map; these seven markers amplified polymorphic PCR products from parental screening (Supplementary file 1; Table S1).

#### Genetic map construction and QTL analysis

A genetic linkage map was constructed using MapDisto 2.0 (Ref^31^). The command ‘Find linkage groups’ was used to construct linkage groups. The minimum log-of-odds (LOD) threshold of 3 and maximum recombination fraction (r) of 0.35 were used to search for linkage groups. The commands ‘Compare all orders’ and ‘Order a linkage group’ were used to compute best order of sequences respectively for linkage groups with short sequences (< 10 loci) and long sequences (> 10 loci). The ‘Sum of adjacent recombination frequencies (SARF)’ option was chosen for ordering sequences. The commands ‘Ripple order’ and ‘Check inversions’ were used to refine the order of sequences generated by ‘Order a linkage group’ command. Double recombinants were removed using ‘Show double recombinants’ command followed by ‘Replace error candidates with missing data’ command. Finally, ‘Bootstrap order’ command with 1000 permutations was used to evaluate the stability or robustness of a given order.

Because many SNPs were very closely linked and did not provide additional information about the QTL locations, we selected only 251 SNPs that were evenly distributed across nine linkage groups for final linkage map construction and QTL mapping. The final linkage map contained 251 SNPs and seven SSRs. Linkage groups were aligned with the pseudo-molecules in the lettuce genome (Genome ID 28333) by BLASTing sequences containing SSRs and SNPs against the lettuce whole genome sequence^32^. The sequences for SSRs were retrieved from NCBI database^33^. The data were used to perform a QTL analysis using QTL Cartographer v2.5 (Ref^34^). Composite interval mapping (CIM) was performed to build an initial model for multiple interval mapping (MIM) procedure. The CIM analysis was implemented using the following criteria: standard model, ‘forward and backward method’ for automatic cofactor selection, a 10-cM window size, automatic selection of 5 control markers, walking speed of 1 cM, and threshold LOD score estimated empirically with 1000 permutations. The MIM analysis was conducted using QTL peaks with empirical LOD threshold value of 2.0 and minimum 5-cM interval between QTL as the initial model. Because the QTL analysis is an iterative process, QTL were searched and refined in a cyclic stepwise fashion using ‘Searching for new QTL’, ‘Testing for existing QTL’, and ‘Optimizing QTL positions’ commands. QTL detected at large marker intervals were deleted as they could be ‘ghost QTL’. New QTL models were only accepted into the current model if they reduced the Bayesian Information Criterion (BIC) values. The epistatic interactions between each pair of QTL were tested using the option ‘QTL interaction’. Non-significant epistatic interactions between QTL were deleted from the model. The model with the least BIC was selected to report the statistically significant putative QTL. QTL effects were estimated using the ‘summary’ option. Sequences for markers in the vicinity of detected QTL were BLASTed^35^ to identify the putative functions of the QTL. The QTL was visualized by MapChart 2.32 (Ref^36^).

## Results

### Chi-square goodness-of-fit test for one-locus and two-locus models

The F_2_ genotypes were deduced based on the F_3_ progeny test. We scored 21 homozygous resistant, 65 heterozygous, and 39 homozygous susceptible F_2_ individuals (Supplemental file 1) of the cross Valmaine × Okeechobee, which resulted in a χ^2^ of 5.38 at two degrees of freedom with two-tailed *P*-value of 0.067 (Table 1). The total number of resistant (combining 21 homozygous resistant and 65 heterozygous) and susceptible individuals in this F_2_ population were 86 and 39, respectively. The χ^2^ of 2.563 at one degree of freedom with *P-*value of 0.10 was obtained for phenotypic data in F_2_ plants.

The heterozygous F_3_ families (65 heterozygotes) produced 541 resistant and 211 susceptible plants based on a progeny test. The χ^2^ of 3.752 at one degree of freedom with a *P*-value of 0.0528. Because the *p* value is greater than α = 0.05, we failed to reject the null hypothesis (Table 1). Thus, results from ${X}^{2}$ test on monogenic dominant inheritance model (Table 1) suggested the likelihood of stippling resistance being controlled by a single dominant allele.

Furthermore, the ${X}^{2}$ test on two-locus model supported (*P* < 0.05) neither complementary gene action (9 resistant: 7 susceptible) nor recessive suppressor (13 resistant: 3 susceptible) (Table 1). Therefore, it is most likely that stippling damage resistance in the romaine lettuce ‘Valmaine’ may be controlled by a single dominant allele, which we named *Stippling damage 1 locus* (*Sd1 locus)*.

### Analysis of Variance (ANOVA) in F_3_ families

The analysis of variance detected significant differences *(P* < 0.0001) among the 125 F_3_ families tested in these experiments (Supplementary file 3). The differences observed indicated the presence of segregation on the F_3_ families from the cross ‘Valmaine’ and ‘Okeechobee’; family 11 was the most significantly (*P* = 0.0117) different F_3_ family when compared to family 10, the most susceptible in these tests. These results were supported by the fact that cultivar ‘Valmaine’ had low stippling damage score (~ 0.4), while Okeechobee had high stippling damage score (~ 3.5) *(P* < 0.0001) (Supplementary file 3). Eleven F_3_ families were reported with less stippling damage, very similar to cultivar Valmaine (*P* > 0.05) but no family was significantly less damaged than this cultivar.

The estimate of *H*^2^ was 0.58 ± 0.09. Likewise, the estimate of *σ*^2^* G* was 1.30 ± 0. The estimates of both genetic parameters were significantly greater than zero based on Z-scores at 0.05 significance level. Raw phenotypic data for F_3_ families are presented in supplementary file 4.

### Linkage map

The seven SSR markers were mapped onto LG2 (LSSA-03b), LG3 (LSSA12), LG4 (LSSA04 and LSSA07), LG5 (LSSA14), and LG6 (LSSB31 and LSSB40). The final linkage map with nine linkage groups spanned a total length of 1056.2 cM, with an average marker spacing of 4.09 cM. In the ‘Valmaine × Okeechobee’ mapping population, LG5 is the longest linkage group with a total length of 355.8 cM, whereas LG9 is the shortest linkage group with a total length of 17.0 cM. The largest marker interval of 42.5 cM exists on LG5 (Supplementary file 1; Fig. 2).

### QTL discovery

The MIM analysis discovered one novel QTL (347.8 cM) on LG5, with LOD score of 3.3 and additive effect of 0.50, which we named *qSTP5 (QTL for stippling damage on Linkage Group 5)*. The peak of QTL *qSTP5* was flanked by SNP188 (343.9 cM) and SNP187 (349.0 cM). The QTL *qSTP5* explained 13.7% of total phenotypic variation for stippling damage suggesting that *qSTP5* had major effect on resistance to stippling damage. The 1-LOD for the QTL is located between 339.03 cM and 352.03 cM (Fig. 1). The resistant parent Valmaine was the source of origin for beneficial QTL allele. The sequences for the markers within the proximity to the QTL are provided in the Supplementary file 1; Table S2.

## Discussion

This is the first attempt to determine the inheritance of the resistance against a devastating pest for lettuce, the serpentine leafminer, *Liriomyza trifolii*. The pest causes economic losses in lettuce production areas in Florida^4^ and California^37^ and it is a concurrent problem in other production areas worldwide^38,39^. *Liriomyza trifolii* in California has at least two biotypes^40,41^. However, no information is available about *L. trifolii* biotypes present in Florida. The crop is limited to field production areas in South Florida; therefore, we believe that there is not too much variability within the insect population. The results presented in this research may be limited to the Florida location as it remains unknown whether there is enough genetic variability in the insect population. Field tests were not conducted due to limitations with infestations and therefore it is not possible to determine whether these results could be extrapolated to natural infestations occurring in the field. Although unknown, light spectrum may not influence feeding behavior of *L. trifolii*; in the present study, some F_3_ families had values of 1, while some had values of 4; if light was affecting feeding behavior, these families would not be as susceptible as it was found in this research.

In the current study, a single dominant allele controlled the resistance to this pest and was named the *Stippling damage 1 locus* (*Sd1 locus*). Likewise, a single dominant allele controls the resistance to *D. balteata* in romaine lettuce cultivar Valmaine^25^. The mapping population ‘Valmaine × Okeechobee’ was developed to study the genetics against both pests, *D. balteata* and *L. trifolii* in two related but independent studies, suggesting that the same locus or two tightly linked loci controlled the resistance to both in romaine cultivar Valmaine.

Both, single locus as well as multiple loci controlling resistance to *L. trifolii* in other crops have been previously described. One major QTL or locus is responsible for resistance to *L. trifolii* in tomato, *Lycopersicon hirsutum*^42^. In contrast, polygenic inheritance of resistance to *L. trifolii* was reported in wildtype tomato, *L. cheesmanii*^43^*.* A single dominant locus is suspected to be involved in the resistance against *L. trifolii* in melon (*Cucumis melo*)^44^. The same is true about other species in the genus *Liriomyza*. A single dominant locus model explained the resistance to a *L. sativae* in melon^45^. However, resistance to *L. langei* in spinach (*Spinacea oleracea*) is a complex trait presumably controlled by several genes with minor effects^46^. Resistance in lettuce to other insect-pests such as *Nasonovia ribisnigri* biotype Nr:0 is controlled by a single dominant allele^47^. However, the genetics against *N. ribisnigri* in lettuce depends on the source of resistance; it has been described as complete to partial depending on lettuce cultivars^48^.

Regardless of how many gene/s were identified in this research, the *Sd1 locus* certainly confers high resistance to insect-pests in cultivar Valmaine. Polarity of chemical constituents of the latex in Valmaine are most likely responsible for its feeding deterrence to multiple insects^49,50^. The latex contains bioactive nitrogenous compounds (most likely glutamic acid) that play a role in the insect resistance through constitutive defense^51–53^; while insect damage elicits induced defense through the production of phenolic compounds^52^ (phenylalanine ammonia lyase, polyphenol oxidase, and peroxidase). Valmaine is an obsolete romaine lettuce cultivar extensively used as a parent in the 1970s through 1980s in the University of Florida Lettuce Breeding Program to develop commercial cultivars^54,55^. The cultivar is susceptible to dieback caused by two soilborne viruses^56^ and resistant to Fusarium wilt of lettuce^57^. Valmaine and Okeechobee share pedigree (Supplementary file 1; Fig. S3), albeit *L. trifolii* resistance was not selected when Okeechobee was developed. Okeechobee was developed by a private seed company using parent cultivar ‘Terrapin’. Valmaine has a high level of resistance to the Diptera *L. trifolii*^2^*,* the Coleoptera *D. balteata*^52,53,58^, and two Lepidopterans; *Trichoplusia ni* and *Spodoptera exigua*^51^, whereas Okeechobee is susceptible to *L. trifolii* and *D. balteata*^4^; it is unknown whether this cultivar is also resistant to *T. ni* and *S. exigua*.

The shared parentage of the population ‘Valmaine × Okeechobee’ may have masked the detection of other minor resistance genes. Although ‘Okeechobee’ is susceptible to *L. trifolii*, there might be resistant loci with small effects present in both cultivars that were masked at this point. Nevertheless, transgressive segregation was not observed indicating that any resistant allele possibly present in Okeechobee’s genetic makeup is not complementary to the ones in cultivar Valmaine. Therefore, future efforts should be in place to determine the existence of other minor resistance loci not identified here.

We initially determined that a single-gene-inheritance model explained the stippling resistance in romaine lettuce. Results from our QTL mapping study identified a novel QTL (*qSTP5*) that controlled stippling damage resistance and was mapped on LG5 (~ 348 cM). The peak of QTL was flanked by SNP188 and SNP187 (Fig. 1). Therefore, gene models as well as QTL analysis suggested that *L. trifolii* resistance in romaine lettuce was a simply inherited trait in mapping population Valmaine × Okeechobee. Likewise, highly significant estimate of broad-sense heritability (*H*^2^) suggested that a statistically significant proportion of phenotypic variance was explained by genetic factors such as allele frequencies; therefore, the trait is heritable and breeding schemes that exploit the genetic variation in cultivar Valmaine should be used. However, the *H*^2^ estimate is moderate (0.58) in the current study and smaller than those previously reported on lettuce against *Liriomyza* spp^22,23^.

The SSR markers LSSB07 (290.7 Mb) and LSSA09 (304.1 Mb) in the vicinity of *qSTP5* were mapped in the lettuce genome^29^. Both markers lie between SNP188 (289.2 Mb) and SNP187 (297.7 Mb) in the ‘Valmaine × Okeechobee’ genetic map and are candidates for subsequent fine mapping. BLAST search of these sequences for the markers closely linked to the QTL showed that the *qSTP5* region clustered leucine-rich repeat receptor-like kinase (LRR-RLKs). These regions belong to a major resistance clusters (MRC5) on LG5^59^. The *qSTP5* probably lies in the resistance gene clusters that could play a role in providing resistance to insect-pest and diseases.

### Implications for breeding

The utility of QTL information presented in the current study could help breeders design a breeding strategy to develop resistant/tolerant cultivars to pests such as *L. trifolii*. Given the high economic importance of the trait for lettuce growers in Florida and everywhere else, the isolation of underlying gene is crucial for developing *L. trifolii* resistant lettuce cultivars. However, this type of research should be replicated with more mapping populations developed from contrasting parents to seek for small effect genes that may have been masked due to the shared genetic relationship between cultivars ‘Valmaine’ and ‘Okeechobee’ (Supplementary ﻿file 1; Fig. S3). Thus, future studies should exploit recombinant inbred line mapping populations derived from parents with wide genetic distance. Saturating the QTL regions with closely linked markers should help breeders in introgression of resistance QTL alleles into commercial lettuce cultivars by marker-assisted selection. Because the stippling damage is most likely a simply inherited trait, the resistance allele from Valmaine should be readily transferrable to elite germplasm. However, multiple loci genetics is not discarded. Development of *L. trifolii* resistant romaine lettuce cultivars should help reduce the dependence of growers on insecticides to control this devastating pest.

## Supplementary information

Supplementary Information 1.

Supplementary Information 2.

Supplementary Information 3.

Supplementary Information 4.

## Data Availability

All data generated or analyzed for this study are included in this published article and its supplementary files.
