# Supplementary material for: Identification and mapping of quantitative trait loci for resistance to Liriomyza trifolii in romaine lettuce cultivar ‘Valmaine’
Source: Sci Rep. 2021 Jan 13;11:998. doi: 10.1038/s41598-020-80050-5 (PMC7807064; doi:10.1038/s41598-020-80050-5)
Supplement: Supplementary file 1 — Supplementary Information 1. [file 41598_2020_80050_MOESM1_ESM.docx]

Identification and mapping of quantitative trait loci for resistance to *Liriomyza trifolii* in romaine lettuce cultivar ‘Valmaine’

Ramkrishna Kandel^1^, Huangjun Lu^2^, Germán V. Sandoya^2*^

^1^ Horticultural Sciences Department, University of Florida, Gainesville, FL 32611 United States

^2^ Everglades Research and Education Center, Institute of Food and Agricultural Sciences/University of Florida, Belle Glade, FL 33430 United States

Ramkrishna Kandel ([rkkandel@ufl.edu](mailto:rkkandel@ufl.edu))

Huangjun Lu (luhuangjun@yahoo.com)

Germán V. Sandoya^2^ ([gsandoyamiranda@ufl.edu](mailto:gsandoyamiranda@ufl.edu))

^*^Correspondence to Germán V. Sandoya ([gsandoyamiranda@ufl.edu](mailto:gsandoyamiranda@ufl.edu))

**Supplementary Materials**

**Supplementary file 1; Figure S1** Subjective rating scale used in experiments to phenotype stippling damage. The rating scale had values of 0 to 4, where 0 = 0-20 stipples, 1 = 21-75 stipples, 2 = 76-150 stipples, 3 = 151- 250 stipples, and 4 > 250.

**Supplementary file 1; Figure S2** Representation of 9 linkage groups of *Lactuca sativa* L. from an intraspecific F_2_ population, constructed with 251 SNPs and seven SSRs. Linkage groups spanned a total length of 1,056.2 cM. Linkage group (LG) 5 is the longest LG, spanning 355.8 cM, while LG9 is the shortest that spanned 17.0 cM. The average marker interval is 4.09 cM and the maximum marker interval of 42.5 cM exists on LG5. Map distances are shown in cM (Kosambi) on the left of each linkage group, while genetic markers are on the right of each linkage group.

**Supplementary file 1; Figure S3** Pedigree relationship between cv. Valmaine and Okeechobee.

**Supplementary file 1; Table S1** SSR primers (Rauscher and Simko, 2013) that are polymorphic in this study.

**Supplementary file 1; Table S2 Sequence information on SNP markers that are in the vicinity of QTL for L. trifolii resistance.**


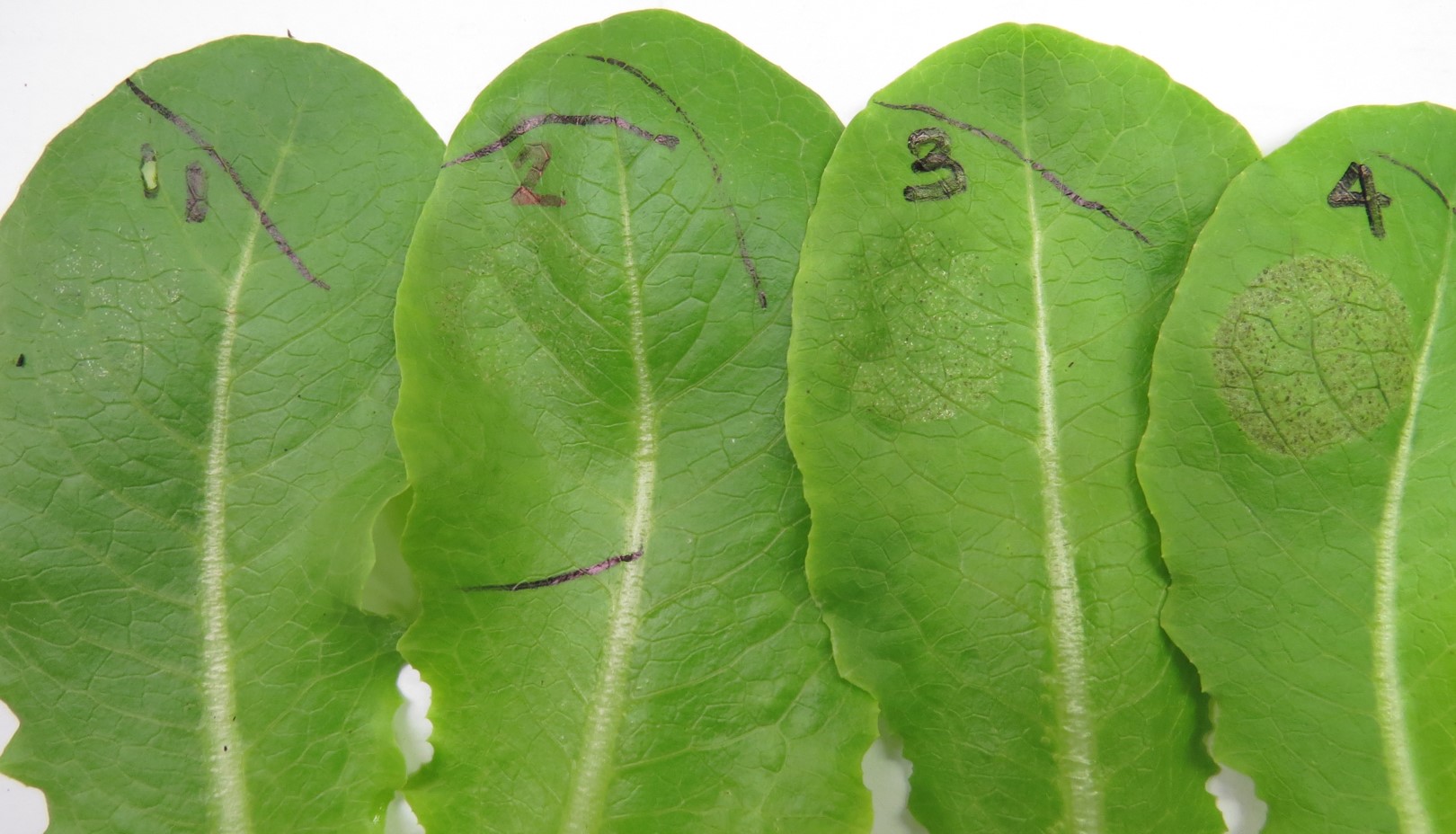


**Supplementary file 1; Figure S1** Subjective rating scale used in experiments to phenotype stippling damage. The rating scale had values of 0 to 4, where 0 = 0-20 stipples, 1 = 21-75 stipples, 2 = 76-150 stipples, 3 = 151- 250 stipples, and 4 > 250.

**Supplementary file 1; Figure S2** Representation of 9 linkage groups of *Lactuca sativa* L. from an intraspecific F_2_ population, constructed with 251 SNPs and seven SSRs. Linkage groups spanned a total length of 1,056.2 cM. Linkage group (LG) 5 is the longest LG, spanning 355.8 cM, while LG9 is the shortest that spanned 17.0 cM. The average marker interval is 4.09 cM and the maximum marker interval of 42.5 cM exists on LG5. Map distances are shown in cM (Kosambi) on the left of each linkage group, while genetic markers are on the right of each linkage group.


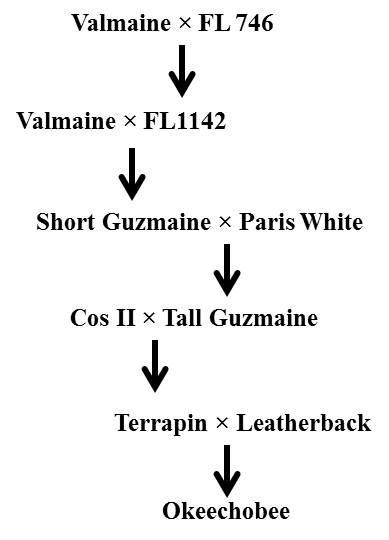


**Supplementary file 1; Figure S3** Pedigree relationship between cv. Valmaine and Okeechobee.

| Marker | Repeat | Copy | Forward primer (5’ – 3’) | Reverse primer (5’ – 3’) | Ann. Temp | Size (bp)^‡^ |
| --- | --- | --- | --- | --- | --- | --- |
| LSSA04 | TC | 14 | AGG AAA GGA AGG GTT GAC TTG T | GGT GAA GAA AAG AGA GAG T | 54 | 224 |
| LSSB40 | CT | 20 | TGG TTT TGG TCC CTG TGA TT | GGT CCG ATA TGT GGT GCT CT | 61 | 227 |
| LSSA12 | GT | 11 | TCG AAA ATT TGG AGA GAG TTT CTT | ACA AGG CCC AAT CCT TTT CT | 61 | 271 |
| LSSA14  LSSB31 | AG  TC | 18  14 | TGA GTT GTA GAG CAA CGA GTG C  TTG GTT TGA CCC CTG TTT TC | CAT GGA AGG TCA TAA GTC CCC  TCA CAC CCA AGC TGT TGC TA | 60  59 | 217  307 |
| LSSA07 | CCA | 5 | TCT GCT TTT AGT GTG TGT CG | GAT ACA CAC AAC CAC CCT TG | 55 | 112 |
| LSSA03b | AAAT | 4 | CTC ACA ACC GAG TCA AAT AAC | TGG GAA TTT TAC CTG ACA AC | 55 | 188 |

**Supplementary file 1; Table S1** SSR primers (Rauscher and Simko, 2013) that are polymorphic in this study.

^‡^ is fragment size amplified by primers before attaching M13-F tail.

**Supplementary file 1; Table S2 Sequence information on SNP markers that are in the vicinity of QTL for L. trifolii resistance.**

| Marker | SNP | Sequence (5’ – 3’) |
| --- | --- | --- |
| SNP192 | G/A | CATGCTTTACAGTAATTTCTCCATTTTCTACACACTCACGAATGAAATGAAATC[G/A]GATATCTATATGTTTGCTTCTTCCATGGAACACTAGATTTTTCATCAAATCTAATGAGGACTTGTTATCCACCAACAGCTCAACTGGAGGCACCTTTTGA |
| SNP191 | G/A | CATGCATGATGTTTTTACAACCAGACGTATAAATAGAAACCCATACTCATCCAGTAACCCTAATTGGCAATTCCGGGC[G/A]ATTTTCAGAGGGTTTCAAGTGAAGAACGCA |
| SNP190 | T/G | CATGCTCTTCACATGGGAATTCA[T/G]CACATCCTGACAGATTCATCGCTTTTTACATATCATCATGACACTAATACTCCATATCTTCTGTTATATGCCAATGACATTGTGCTCACCGCCGCCTCCA |
| SNP189 | A/C | CATGCCACTTAAATTCTCTTCACACCTA[A/C]TTGTTCAACAGACCACATTCTGGTGTCAACAGTAATATAAAACACAATAATGAATCAAAATATGTGTGAATTCTCCTCACTCTTCGACA |
| SNP188 | C/T | CATGTTCAAAAGTCGGGAAAGCCCTAGTTGACTCG[C/T]CGAGTCTTCGTGCTGACTTGCCGAGTCCATGTAGAAATCCTTGCACGACAATCTTCACTGGACTCGCCGAGTTGGCCATGCAACTCGTCGAGTCCCTTAG |
| SNP187 | T/C | CATGCGCATTGGTGAGCCAAAATGGCATCACCACGAACTCGTAATGCCAATAACGAGTC[T/C]GGAACGCGGTCTTCTCCACGTCCTCCTCCCTCACTCTAACATGATAATACCCAGA CCTCAC |
| SNP186 | G/T | CATGCTAACA[G/T]CTTGTGTGACCGACTTTGGTAAAGCATGGGATATCCATTTACCATTGGTCGAGTTTTCATACAACAACAACTACCATACTAGTATCAAAGTTTCTCCATT |
| SNP185 | A/G | CATGTGTTATTAAGTTCGGAAAGTTGTGGGACACACATTTGCCTTTTATTAAGTTCTCGTGTAGCAACAGCTATCACACCAGTATCAAGACTGCTCC[A/G]TTCGAAGCCCTCTACCGGTTATAAGTACA |
